# Supplementary material for: Patterns of Intron Gain and Loss in Fungi
Source: PLoS Biol. 2004 Nov 30;2(12):e422. doi: 10.1371/journal.pbio.0020422 (PMC532390; doi:10.1371/journal.pbio.0020422)
Supplement: Table S1 — Also available at http://genes.mit.edu/NielsenEtAl/. (4.3 MB ZIP). [file pbio.0020422.st001.zip › NielsenEtAl/html/247.html]

AN4871.1.NCU04554.1.MG07927.1.FG10939.1


```
 CLUSTAL W (1.82) Multiple Sequence Alignments - Introns Inserted


Sequence 1: NCU04554.1	575 aa
Sequence 2: FG10939.1	399 aa
Sequence 3: MG07927.1	396 aa
Sequence 4: AN4871.1	398 aa
Alignment Length: 584 aa
Number Identitical Residues: 199 aa
Alignment Score (without introns) 10437


MG07927.1 	-------~----------------------------------------------------
NCU04554.1	MTASRHI1LYATALNQTTKTRRIRIAKQSHEKTPINGQTPPPRAFSPSLTSPFVHLSPES
FG10939.1 	-------~----------------------------------------------------
AN4871.1  	-------~----------------------------------------------------
          	                                                            

MG07927.1 	--------------------------------------------------~---------
NCU04554.1	QVEQSISSAPSRLLRRIEVHLHRILSVPLGPAFCNPRNPKGCAIVCHPHQ0LYSGHQHPT
FG10939.1 	--------------------------------------------------~---------
AN4871.1  	--------------------------------------------------~---------
          	                                                            

MG07927.1 	------------------------------------------------------------
NCU04554.1	ATIATTATVTFTESVTATITSVDKGPTPSSVTSTTPATTETSTTTTEQQTTPTSPTMGGG
FG10939.1 	--------------------------------------------------------MGGG
AN4871.1  	------------------------------------------------------------
          	                                                            

MG07927.1 	---MTQRTVAYFVNW~AIYGRKHRPQELPADKLTHVLYAFANVKPDSGEV~FLTDTWADT
NCU04554.1	PDTTGYRTVAYFVNW~AIYGRKHRPQDLPAEKLTHILYAFANVRPDSGEV~YLTDQWADT
FG10939.1 	PE--GFRTVAYFVNW2AIYARKHRPQDLPVENLTHILYSFANIRSDSGEV2HLTDSWADT
AN4871.1  	--MSGYKTVGYFVNW0AIYGRNYNPQDLPAEKLTHILYAFANVRPETGEV2YLSDTWSDI
          	      :**.***** ***.*::.**:**.::***:**:***::.::*** .*:* *:* 

MG07927.1 	DIHWEGDSWNDEGTNLYGCLKQLNLLKKANRNLKVLLSIGGWTYSSNFRAPASTPEGRSH
NCU04554.1	DIHWEGDSWNDSGTNLYGCLKQLNLLKRRNRNLKVLLSIGGWTYSSNFKEPASKEEGRRR
FG10939.1 	DIHWDGDSWNDVGTNLYGCMKQLNLLKRRNRNLKVLLSIGGWTFSSNFKGPASTPQGRAT
AN4871.1  	EKHYPTDSWNDTGNNVYGCVKQLGLLKRQHRQLKVLLSIGGWTYSPNFTNGAGTPENRAR
          	: *:  ***** *.*:***:***.***: :*:***********:*.**   *.. :.*  

MG07927.1 	FARTAVEL~VRQLGFDG~IDIDWEYPQN~PQEAADLVALLQACRH~ELDAYAHSVDGENP
NCU04554.1	FAESAVEL0VKNLGFDG~LDIDWEYPQN~AQEASDFVSLLSACRS~ALDAYASTVPG--N
FG10939.1 	FAKSCVDL~IKNLGFDG~IDIDWEYPQN~ADEARNYVELLGAVRY~EMDAYAQTLSR--P
AN4871.1  	FAQTATKL~ITDLGFDG1IDIDWEYPQN1DQQAQNYVDLLRRCRE0ALNAAQG------Q
          	**.:...* : :***** :*********  ::* : * **   *   ::*          

MG07927.1 	YHFELTVACPAG~ASNYEKMDIAGMDPLLDFWNLMAYDYAGSWDQTAGHQANLFPSHGNP
NCU04554.1	PHFEMSVACPAG~PQNYEKMDLPGMDRYLDFWNLMAYDYAGSWDQVAGHQANLFHCNSNP
FG10939.1 	YHFELTVACPAG~ATNFQKLDIRGMDRYLDFWNLMAYDYAGSWDQTAGHQANLYPSHDNP
AN4871.1  	RRFQLTVAVPAG1PDNYNKLRLQEMTPYLDFYNLMAYDYAGSWDQTAGHQANLYPSTSNP
          	 :*:::** *** . *::*: :  *   ***:*************.*******: . .**

MG07927.1 	TSTPFSTDAAIAHYASQG-VALDKIVMGMPIYGRAFENTDGPGAPFQGVGQGTWEQGVYD
NCU04554.1	AATPFSTQRAVEYYLSTG-VAADKIVLGMPLYGRAFQGTEGLGQPYSGVGEGTWENGVHD
FG10939.1 	VSTPFSTSAAIDFYVRSG-VSPSKIVLGMPLYGRAFQNTDGPGRPYQGVGEGTWEQGVYD
AN4871.1  	TSTPFNTVQAVNHYIDAGGVPSNKIILGMPIYGRAFQNTDGPGRPYSGIGQGTWEQGVYD
          	.:***.*  *: .*   *.*. .**::***:*****:.*:* * *:.*:*:****:**:*

MG07927.1 	FKALPLPGAEERFDEEAGATYSYDPATRHMVTYDTVPLARRKAEFIRERGLGGAMWWESS
NCU04554.1	YKKLPLEGAEERVDYESGATYCYHPGTKTLVSYDTPGMARMKAEYIRERGLGGAMWWESS
FG10939.1 	YKALPLEGAQEYGDRGCCASYCYNPQTRTMVTYDTPRVAWDKAEYVRKWKLGGAMWWESS
AN4871.1  	YKALPRPGATEQLDTNIGASWSYDPSSREMVSYDTVAAADLKAAYIQSRRLGGAMWWETS
          	:* **  ** *  *    *::.*.* :: :*:***   *  ** :::.  ********:*

MG07927.1 	ADKEG------DESLIGNVVDVFGGPEGLKHQENCLAYPQTKYDNLRNGFPNH
NCU04554.1	ADKEG------PDSLIGTVVEGLGGLPALRWDGNCIDYPNTKYDNLRAGFPGN
FG10939.1 	ADKEG------EKSLITTVVNGFGGQGALMRQDNCIEYPATKYDNLRDGFPNN
AN4871.1  	ADKGGKTANKADGSLIGTFVEDVGGVNNLDRTQNAISYPDSQYDNLKAGFPSS
          	*** *.::..:  *** ..*: .**   *    *.: ** ::****: ***.
```
